# Supplementary material for: Dependence of Successful Airway Management in Neonatal Simulation Manikins on the Type of Supraglottic Airway Device and Providers’ Backgrounds
Source: Children (Basel). 2024 Apr 28;11(5):530. doi: 10.3390/children11050530 (PMC11119467; doi:10.3390/children11050530)
Supplement: Supplementary file 1 [file children-11-00530-s001.zip › Supplemental Figure S1. Instruction for LMA.pdf]

インターサージカル ラリンゲルマスク Solus 簡易取扱説明書

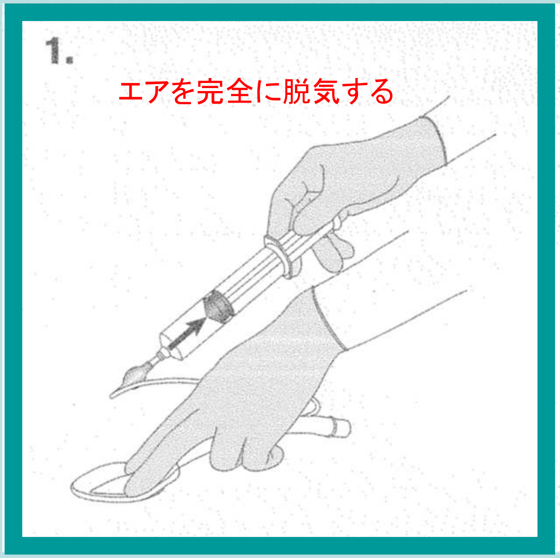

①本品を平面上に置き、シリンジなどでカフから
エアを完全に脱気する。
本品取扱の際は手術用グローブをご使用下さい。

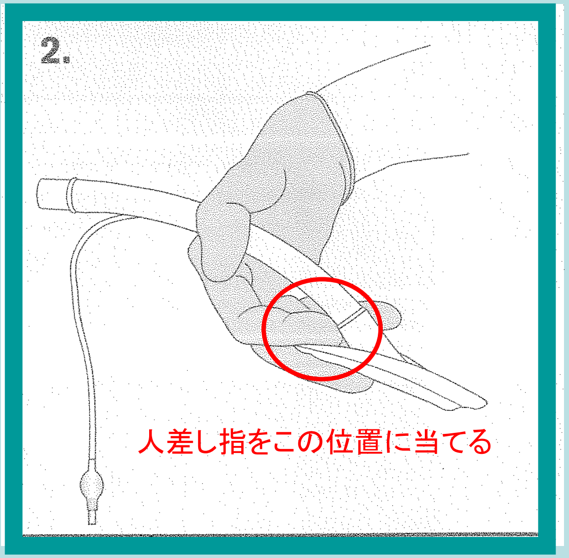

②カフ背面に水溶性潤滑剤を均一に塗付する。
チューブとカフの間の接合部に人差し指の先を
当てて挿入の準備をする。

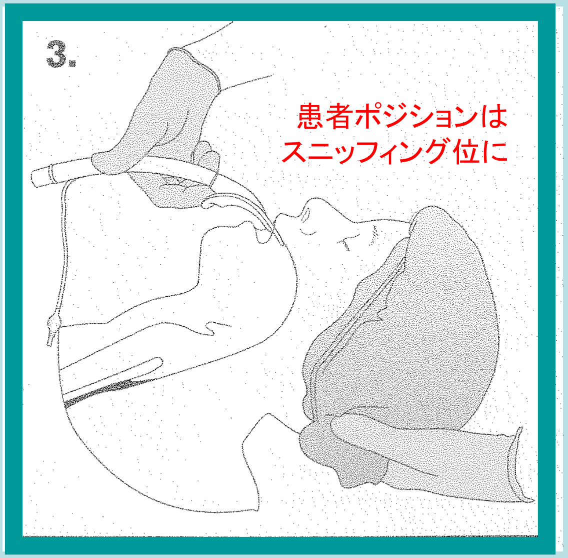

③患者の頭部はスニッフィング位にする。
カフ先端の折れ曲がりに注意しながら、
カフ背面を硬口蓋に軽く押し当てる。

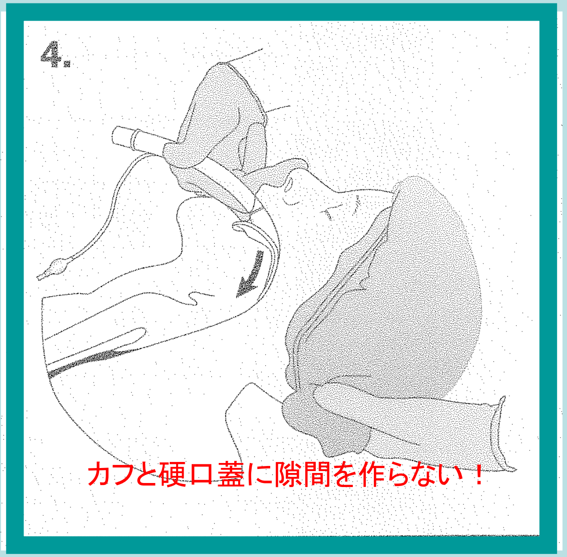

④カフを押し当てたまま、人差し指を硬口蓋から
軟口蓋へと沿わせて、抵抗を感じるまでカフを
挿入する。

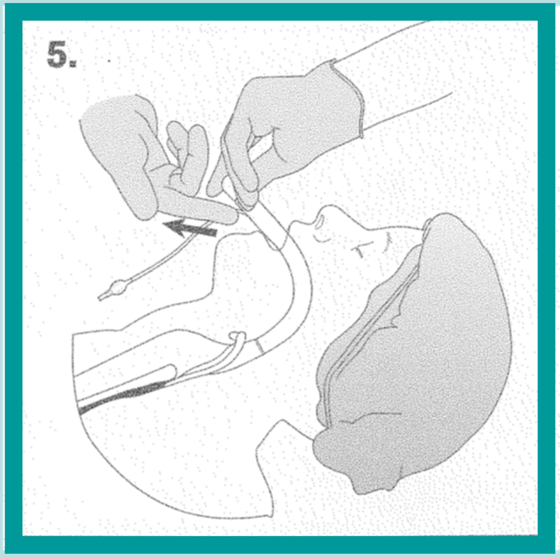

⑤別の手でカフの位置を保持しながら
人差し指を放す。

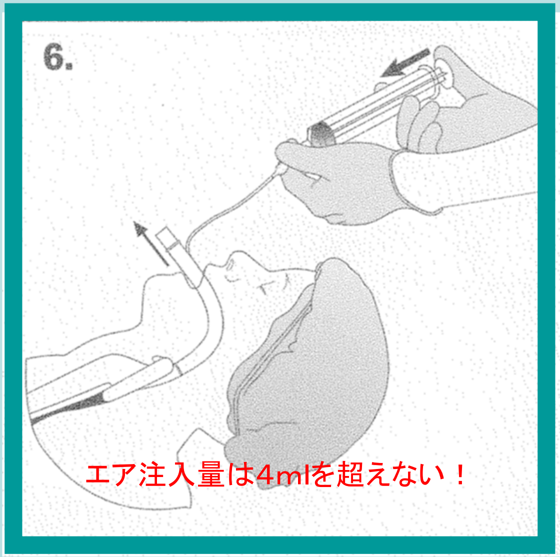

⑥カフにエアを注入する。この時エアウェイ
チューブに表示された推奨値（＜4ml）を
超えて注入しないこと。通常は推奨値以下で
適切に閉塞できる。

※本資料は簡易取説です。使用時には必ず添付文書をご参照いただけますようお願い致します。本品は単回使用品です。

Solus 使用上の注意点

ご使用時には下記の点にご注意下さい。

・挿入時の患者ポジショニングはスニッフィングポジションです。

・水溶性潤滑剤は“カフの背面”にのみ塗付して下さい。
注意：カフ開口部（表面）への塗付は、潤滑剤の気道への落ち込みや、気道反射を誘発する可能性がありますので、絶対に行わないで下さい。

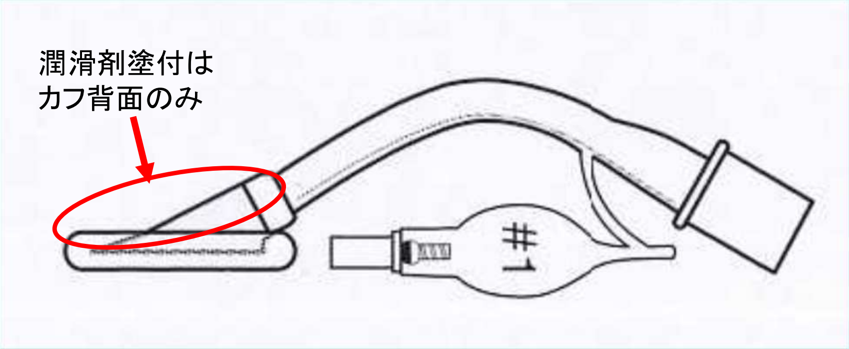

・サイズ1のカフ注入量は4ml未満です。
準備するシリンジは小さめのサイズを選択して下さい。

・挿入時はカフを押し当てたまま進めてください。カフと硬口蓋に隙間が出来ると、カフ先端部がめくれ返って、挿入困難になったり、挿入後にカフが適切に膨らまない可能性があります。
