# Supplementary material for: Dependence of Successful Airway Management in Neonatal Simulation Manikins on the Type of Supraglottic Airway Device and Providers’ Backgrounds
Source: Children (Basel). 2024 Apr 28;11(5):530. doi: 10.3390/children11050530 (PMC11119467; doi:10.3390/children11050530)
Supplement: Supplementary file 1 [file children-11-00530-s001.zip › Supplemental Figure S2. Instruction for i-gel.pdf]

i-gel（小児用）　簡易取扱説明書

＊本品は単回使用品です.

①

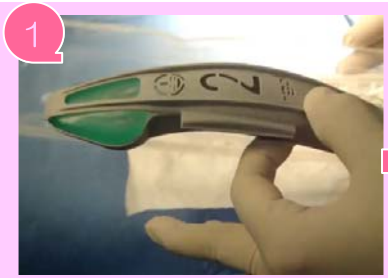

パッケージを開封し，保護ケースをパッケージから取り出します.

本品取り扱いの際は，手術用手袋をご使用下さい.

②

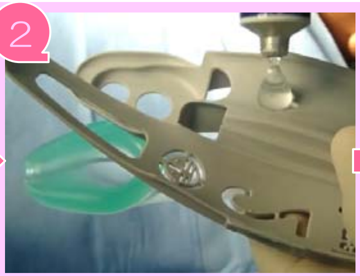

i-gelを取り出し、保護ケースの内側中央部に水溶性の潤滑剤（KYゼリーなど）を出します.

③

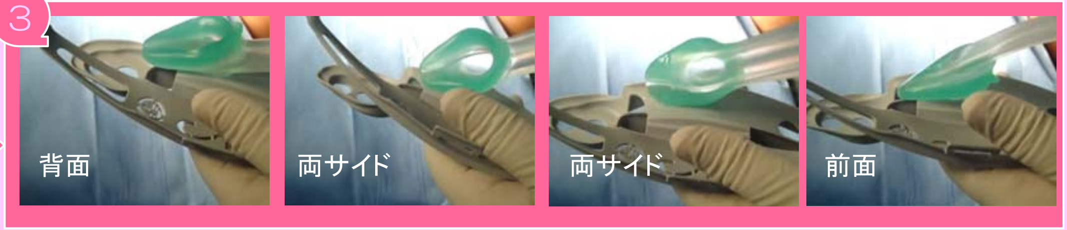

i-gelのバイトブロック部を掴んでカフの“背面”と“両サイド”および“前面”に潤滑剤を均一に塗布します．詳細は裏面をご参照下さい.

---

④

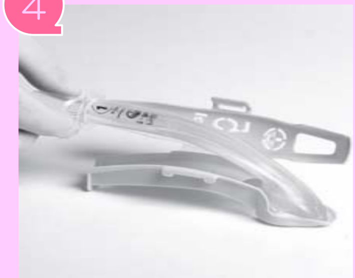

挿入のタイミングまでi-gelを保護ケースに戻しておきます.

注：潤滑剤塗布後の長時間放置は避けるようにしてください。

⑤

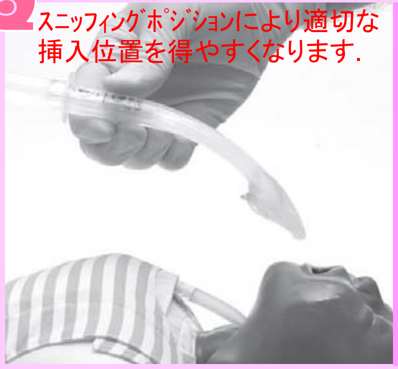

保護ケースからi-gelを取り出し，バイトブロック部をしっかり握ります．その時カフ部の開口面が患者の顎の方を向くようにします.

挿入時のポイント

患者の頭部を後屈させ首を真っ直ぐ後ろに反らせて（スニッフィングポジション）顎を優しく押し下げた状態を保持します.

硬口蓋に向かい患者の口腔内にカフ先端より挿入します.

注：保護ケースはイントロデューサーではありません．患者に挿入しないで下さい.

⑥

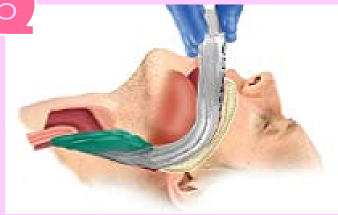

硬口蓋に押し当てる要領で，抵抗を感じるまでi-gelを挿入します．挿入過程では患者の口腔内に指を入れないで下さい.

注：挿入時は無理な力を加えずゆっくりと優しく操作して下さい。

---

⑦

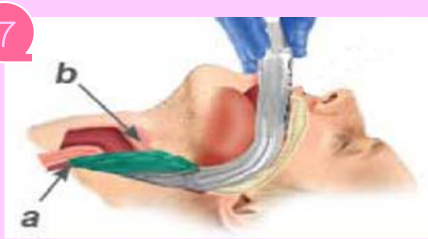

気道確保時には，i-gel先端は食道開口部に（a），カフは喉頭周辺（b）に位置します.

注：小児については患者ごとに挿入長が異なりますのでご注意下さい。

⑧

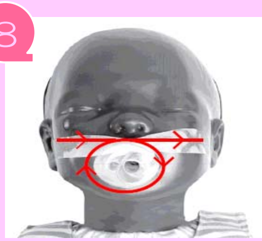

矢印の方向にテープで固定します．（頬骨→上唇側→下唇側→頬骨）

テープで固定するまではi-gelを保持してください．（手を離すと抜けてきたり、適正位置からずれることがあります）

⑨

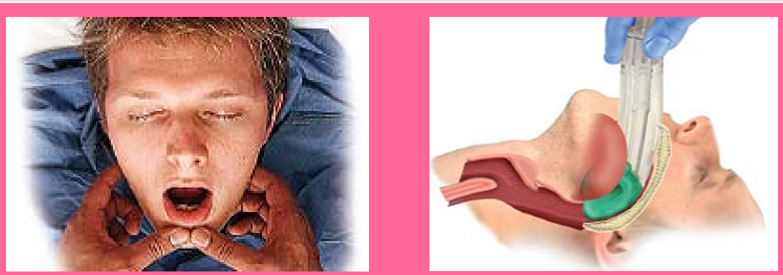

気道確保位置に到達する前に抵抗を感じた場合，用手的気道確保（頭部後屈，下顎挙上法）や，カフを軽く回転させながら挿入することをお勧めします.

※本資料は簡易取説です．使用時には必ず添付文書をご参照いただけますようお願い致します.

＜i-gel 挿入時のポイント＞

①水溶性ゼリーを背面・両サイド・前面にしっかり塗布する
（適切な部分にしっかり塗布しないと，うまく挿入できない恐れがあります．）
②スニッフィングポジションをとり，抵抗を感じる深さまで挿入する
（浅めに挿入しますと，リーク等が発生する恐れがあります．）

～ 水溶性ゼリーの塗布位置と手順について ～

水溶性ゼリーを塗布する場所は下記の通りです．
確実に塗布して下さい．挿入時の抵抗が比較的
少なく，適切な位置に挿入しやすくなります．

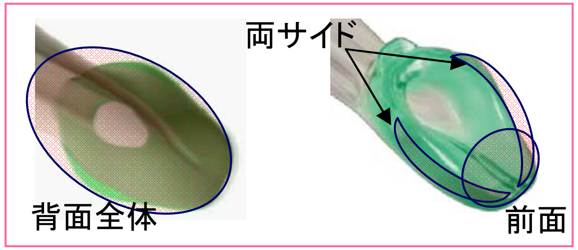

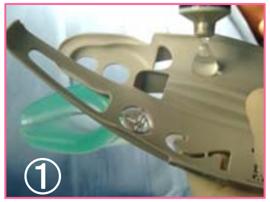

保護ケースに
水溶性ゼリー
（KYゼリーなど）
を適量落とします．

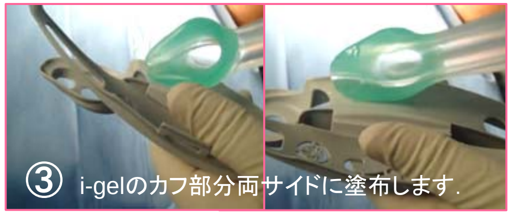

i-gelのカフ部分両サイドに塗布します．

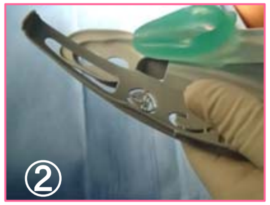

i-gelの背面全体に
塗布します．

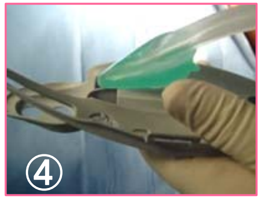

i-gelのカフ部分
前面に塗布します．

～ 挿入時の注意点 ～

挿入時にスニッフィングポジションを取ることで適切な位置（抵抗
を感じる位置まで）挿入しやすくなります．挿入に抵抗を感
じる場合，下記のように用手的気道確保（A）を行うか，i-gel
を軽く回転させながら挿入する方法（B）をお勧め致します．
注：挿入時は無理な力を加えずゆっくりと優しく操作して下さい。

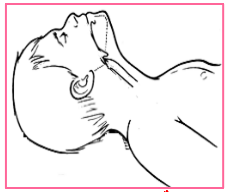

スニッフィング
ポジション

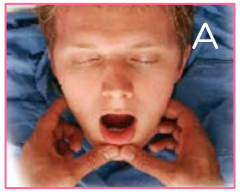

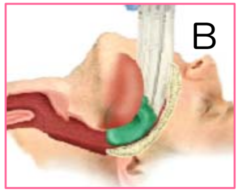

～ 胃管チューブ挿入時の注意点 ～

①胃管チューブ挿入前に必ず胃管チューブ挿入口に水溶性
ゼリーを適量塗布して下さい．
②胃管チューブ挿入時には4～5回チューブを出し入れしながら
挿入するとスムーズに 挿入できます．
＊チューブに直接塗布しない方がスムーズに挿入できます．
注：Size1には胃管挿入ポートはございません。

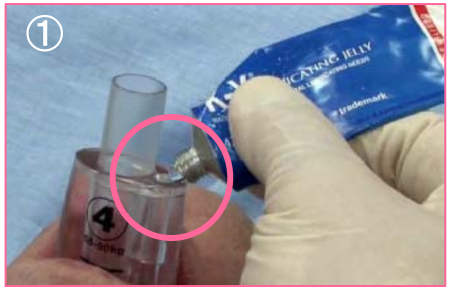

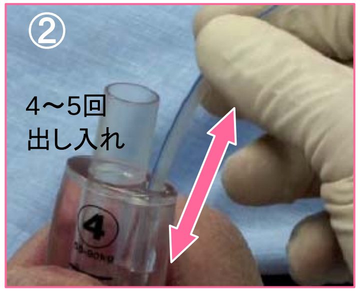

患者体重と i-gel ｻｲｽﾞ の目安

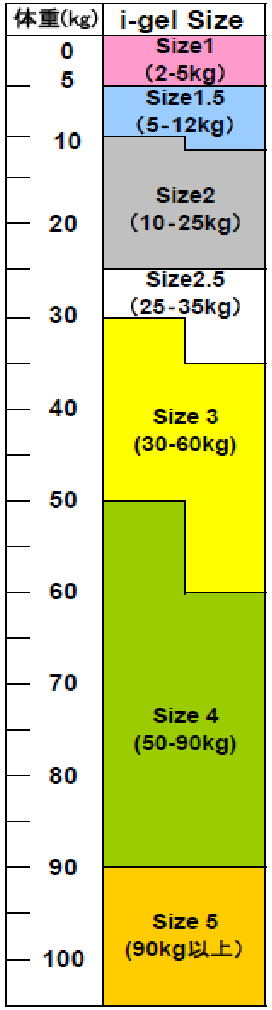

※本資料は簡易取説です．使用時には必ず添付文書をご参照いただけますようお願い致します．本品は単回使用品です．
